# Editorial: Systemic markers of muscle loss

**DOI:** 10.3389/fnut.2023.1210976

**Published:** 2023-06-20

**Authors:** Amanda Soares Santos, Brandon M. Roberts, Gabriela Salim de Castro

**Affiliations:** ^1^Departamento de Radiologia e Oncologia, Faculdade de Medicina da Universidade de São Paulo, São Paulo, Brazil; ^2^Department of Cell, Developmental and Integrative Biology, University of Alabama at Birmingham, Birmingham, AL, United States; ^3^Cancer Metabolism Research Group, Departamento de Cirurgia e LIM 26-HC, Faculdade de Medicina da Universidade de São Paulo, São Paulo, Brazil

**Keywords:** skeletal muscle, muscle loss, cachexia, sarcopenia, aging

Skeletal muscle is indispensable for fundamental physiological processes, including respiration, nutrient acquisition, locomotion, and the regulation of glucose, amino acid, and lipid homeostasis. In adults, skeletal muscle contributes up to 40% of resting metabolism and accounts for a higher percentage of body weight (1, 2). Furthermore, the remarkable adaptability of skeletal muscle is reflected in its capacity to undergo changes in its size, composition, biochemical and metabolic properties. These modifications occur in response to physiological stimuli, including mechanical load and nutrient availability, and pathological stimuli such as cancer and aging (1). Adaptations that transpire in muscles during certain diseases may result in alterations in systemic markers that can serve as biomarkers or predictors of mortality and morbidity (2).

Despite ongoing research, the etiology of skeletal muscle wasting remains incompletely understood, as multiple molecular pathways may contribute to this condition. In this Research Topic, the focus is on elucidating the current knowledge regarding biomarkers, clinical signals, and the effects of diet and physical exercise on skeletal muscle loss. Greater insight into the interplay among these factors is necessary to mitigate muscle loss in both pathological and physiological contexts, such as cancer or aging. The current Research Topic highlights 11 publications addressing these aspects.

Wu et al. describe, in their review, the multifactorial pathways that can lead to muscle loss, as it can encompass aspects such as nutrition, neuromuscular junction damage, dysfunction of satellite cells, disrupted autophagy, systemic inflammation, muscle's reduced protein synthesis, and increased protein degradation through the ubiquitin-proteasome system.

Insulin resistance (IR) is considered an important contributor to several metabolic diseases and recent studies have shown that low skeletal muscle mass is a crucial factor for IR-related diseases such as non-alcoholic fatty liver disease (NAFLD). Guo et al. demonstrated that muscle mass index is negatively correlated with the severity of fatty liver and liver fibrosis in Chinese adults with NAFLD, even if the individuals do not have overt sarcopenia. Knowing this, the authors suggest that interventions to increase muscle mass can prevent or help to control NAFLD progression.

Ding, Guo et al. and Ding, Lv et al. in two articles, seek to find simpler, more economical, and more accurate biomarkers. In one of the articles, Ding, Lv et al. suggest that, due to the increase of systemic inflammation during the cancer cachexia syndrome, the systemic inflammatory immunity index (SII), based on the count of neutrophils, lymphocytes, and platelets in the peripheral blood, combined with the prognostic nutritional index (PNI), calculated by peripheral blood albumin and lymphocyte count, can predict sarcopenia. In this work, a higher SII-PNI score is associated with worse overall survival and disease-free survival at 5 years follow-up. In a second study, Ding, Guo et al. suggest that the ratio of serum creatine (Cr) and cystatin C (CysC) can be used in the screening of sarcopenia in patients with gastrointestinal stromal tumors since it is positively related to muscle mass and handgrip strength. Recurrence-free survival (FRS) was significantly lower in the low serum Cr/CysC ratio group compared to the high ratio group.

Jiang et al. also explored the relationship between prognostic nutritional index (PNI) and overall survival in patients with nasopharyngeal carcinoma (NPC). The study demonstrated that PNI can be a new prognostic factor for patients with NPC. Shi et al. sought to validate the ability of creatinine-to-cystatin C ratio (Cr/CysC) to assess muscle mass and predict a poor prognosis in healthy American adults. The Cr/CysC showed a good performance in identifying cases of low muscle mass and in association with the risk of mortality. In the study, optimal cutoff values for Cr/CysC were < 1.0 in men and < 0.8 in women.

In addition to cancer, sarcopenia is also a risk factor for other diseases. As the diagnosis of sarcopenia requires specific equipment and specialized training, finding circulating biomarkers could be a useful tool when these types of equipment and/or trained personnel are not available. Deng et al. demonstrated for the first time that serum growth differentiation factor (GDF)-15 is associated with sarcopenia in patients with chronic obstructive pulmonary disease (COPD), showing that patients with COPD and sarcopenia presented higher levels of circulating GDF15.

In 2019, the outbreak of coronavirus disease 2019 (COVID-19) spread rapidly around the world and its relationship with sarcopenia has been the subject of several studies. The systematic review and meta-analysis of Xu et al. showed that the overall prevalence of sarcopenia among patients with COVID-19 was 48.0% and could reach 69.7% in patients admitted to the ICU. These results were based on 21 studies involving 5,407 patients with COVID-19. Furthermore, the study demonstrates that COVID-19 survivors, especially older patients, are at greater risk of developing acute sarcopenia. These findings demonstrate that improved monitoring of patients at risk of sarcopenia is crucial, as presence of sarcopenia is associated with low survival (3).

Computed tomography can help in the diagnosis of sarcopenia, and the established method in Western countries is the psoas muscle mass index measured at the third lumbar vertebra (L3-PMI, cm^2^/m^2^), however, the values L3-PMI cut-off values for diagnosing sarcopenia in Asian populations remained unknown. Kong et al. sought in their work to establish appropriate L3-PMI reference values to define sarcopenia in the population of northern China. The group defined the age-specific low skeletal muscle mass reference values by assessing the L3-PMI through CT images of 1,787 healthy subjects aged between 20 and 88 years. The values can be found in Table 3 of the article.

At the end of this journey through the main problems arising from the loss of muscle mass, two articles show us how skeletal muscle is a dynamic tissue. Due to its high regeneration capacity, it is possible to minimize the effects of low muscle mass through a healthier life, which must include regular physical exercises and an adequate diet. However, the aged muscle often no longer responds in the same way, and therefore, nutritional supplementation can be a key element in combating sarcopenia.

Franzke et al. show in their work that a protein-rich diet combined with physical exercise can improve body composition in the elderly, in addition to improving other aspects, such as the innate immune system, lipid transport, and the coagulation system, possibly decreasing the chance of developing chronic diseases. Whether supplementation with fermented oyster extracts would improve sarcopenia was investigated by Lee and Lee. Oyster extract is rich in taurine phosphorus, essential minerals, and glutamic acid, which is a precursor of gamma-aminobutyric acid, which stimulates the release of growth hormone and insulin-like growth factor-1. Supplementation alone did not improve muscle function, while benefits occur through combination with physical activity and physical exercise alone, as the trained groups had their muscle function remarkably improved.

The studies and reviews discussed above offer valuable insights into the implications of skeletal muscle wasting, its diagnostic criteria, and potential interventions. Furthermore, circulating biomarkers are becoming a promising means of diagnosing and monitoring muscle loss. Given the association between muscle wasting and increased morbidity and mortality, particularly among those with chronic diseases and the elderly, the exploration of muscle wasting across various disease states remains a major area of investigation.

## Author contributions

AS summarized the studies of the Research Topic. GC and BR critically reviewed the text and wrote the introduction and the conclusion. All authors contributed to the article and approved the submitted version.

## References

[1] Minetto MA Busso C Gamerro G Lalli P Massazza G Invernizzi M. Quantitative assessment of volumetric muscle loss: dual-energy X-ray absorptiometry and ultrasonography. Curr Opin Pharmacol. (2021) 57:148–56. 10.1016/j.coph.2021.02.002 33735662

[2] Sartori R Romanello V Sandri M. Mechanisms of muscle atrophy and hypertrophy: implications in health and disease. Nat Commun. (2021) 12:330. 10.1038/s41467-020-20123-1 33436614 PMC7803748

[3] Pinto F Andrade M Gatti da Silva G Faiad J Barrére A Gonçalves R. Function over mass: a meta-analysis on the importance of skeletal muscle quality in COVID-19 patients. Front Nutr. (2022) 9:837719. 10.3389/fnut.2022.837719 35529467 PMC9067541

